# Fabrication of zein nanofibrous scaffold containing *Scrophularia striata* extract for biomedical application

**DOI:** 10.1186/s13036-025-00486-z

**Published:** 2025-02-11

**Authors:** Yasin Salahshour, Saadat Rastegarzadeh, Hossein Motamedi, Elham Hoveizi

**Affiliations:** 1 Department of Chemistry, Faculty of Science, Shahid Chamran University of Ahvaz, Ahvaz, Iran; 2 Department of Biology, Faculty of Science, Shahid Chamran University of Ahvaz, Ahvaz, Iran; 3 Biorefinery Research Center, Shahid Chamran University of Ahvaz, Ahvaz, Iran

**Keywords:** Zein, *Scrophularia striata*, Wound healing, Nanofibers, Electrospun scaffolds

## Abstract

Skin wounds have the potential to rapidly become infected, with bacteria having the ability to quickly penetrate to the skin’s deeper layers. Then they enter the lymph nodes and spread throughout the body; therefore, all wounds should be cleaned and have a permanent cover. Modern wound dressings with effective antibacterial and therapeutic properties are required to create a sterile environment for the acceleration of healing. The aim of this work was to prepare zein electrospun nanofibers containing *Scrophularia striata* extract for wound healing promotion. Electrospun nanofibers made of zein, a natural polymer, have attracted a lot of attention due to their biocompatibility and biodegradability. The prepared nanofibers were characterized by scanning electron microscopy (SEM), energy dispersive X‑ray analysis (EDX), water contact angle test, and Fourier transform infrared spectroscopy (FT-IR). The parameters affected by the electrospinning process were investigated and optimized. The results revealed that the zein nanofibers (25% w/v, zein) containing *Scrophularia striata* extract (6.7% w/v) had a smooth and bead-free morphology with improved surface hydrophilicity. The measurement of water contact angle confirmed that nanofibers containing extract showed higher wettability (64.9°) compared to fibers without extract (119.8), so the proposed mat adequately moisturizes the wound environment. The antimicrobial studies show that *Scrophularia striata* extract incorporated nanofibers has the ability to inhibit the growth of both gram-negative and gram-positive bacteria. The biophenols release profile indicated that nanofibrous mat can release more effective substances to promote wound healing. The biocompatibility and biodegradability of nanofibrous scaffold containing *Scrophularia striata* extract tested in in vivo and in vitro conditions show a significantly higher survival rate of fibroblast cells. In addition, macroscopic and histological observations confirmed that the implanted nanofibers containing the extract did not exhibit any signs of inflammation or redness after a month when inserted beneath the skin of mice surrounded by vessels containing epidermis.

**Graphical Abstract:**

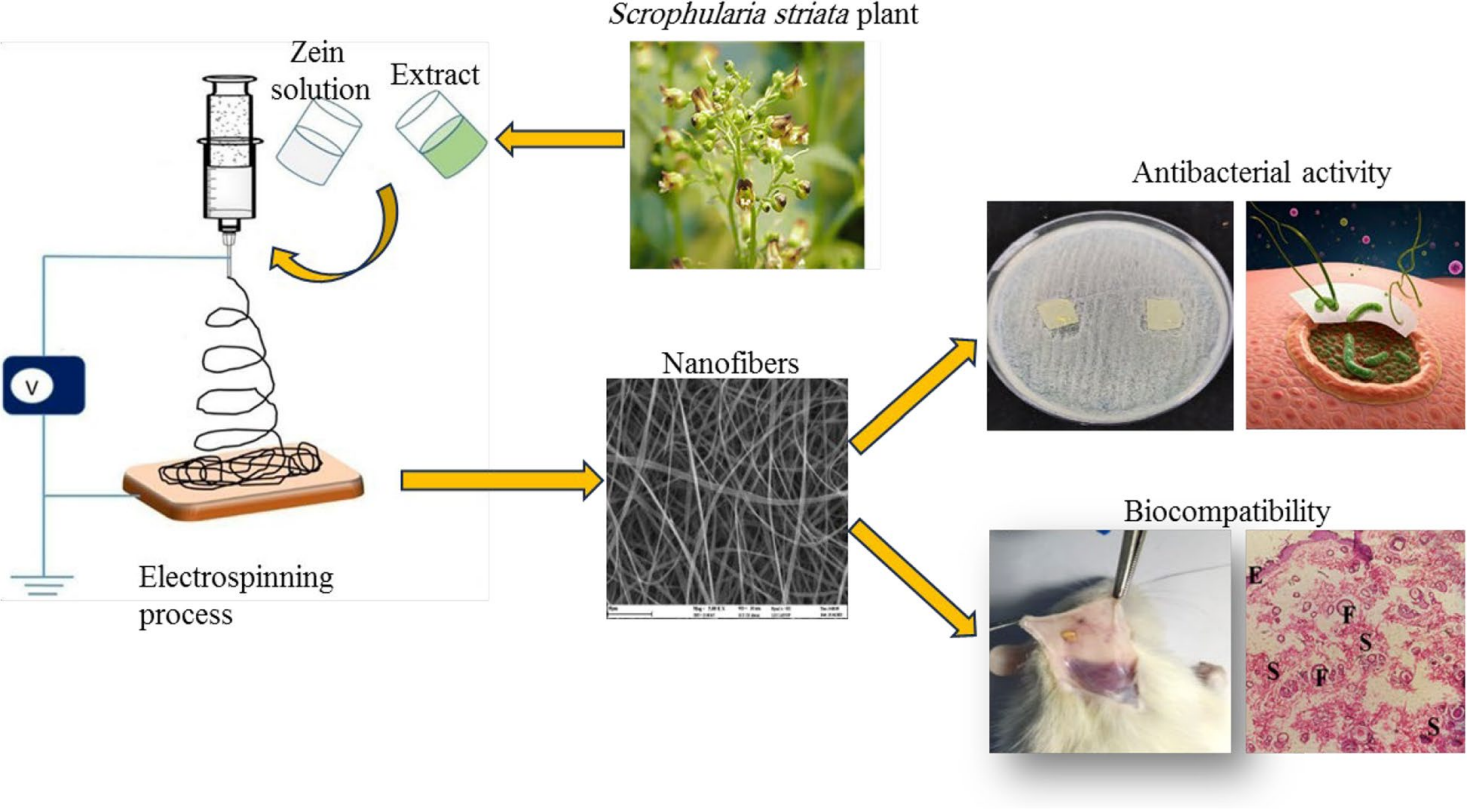

## Introduction

A large number of studies have investigated the inhibitory effects of natural substances against microorganisms. These studies suggest that it is essential to use compounds that are non-toxic to humans and free of side effects. Plant extracts are commonly used for their antibacterial and antioxidant properties [1]. *Scrophularia striata* is one of the most important medicinal plants of the monkeyflowers. This family comprises three subfamilies, 220 genera and 3000 species in the world and has 5 species in Iran [2]. *Scrophularia striata* grows wild and is found in mountain slopes, meadows, and chilly alpine areas like Iran’s Zagros Mountains. The decoction of this plant is traditionally used to treat superficial and deep infections. Washing with a cooled decoction of this plant or its steam has a tremendous effect on treating inflammation and infection, skin burns, pain and inflammation, and ear infections [2].

Nanofibers are relatively short strands with a diameter of less than 1000 nanometers that are created as a single layer on a plane. Reducing the diameter of fibers to the nanometer scale induces intriguing properties, such as a significantly high surface area-to-volume ratio, pronounced porosity, versatile surface functionalization, and outstanding mechanical characteristics encompassing hardness and elasticity [3]. Electrospinning is widely regarded as the predominant method for fabricating nanofibers due to its cost-effectiveness, simplicity, and ease of implementation [4, 5]. The produced fibers’ diameter ranges from tens of nanometers to several micrometers using this technique [6, 7]. One of the significant uses of nanofibers is tissue engineering, which seeks to restore the normal functions of tissues and organs [8]. Nanofibers are also used in the healing of wounds. Biocompatible nanofibers as dressings imitate the extracellular matrix of the tissue microenvironment and offer a favorable surface for cell attachment. To create an effective dressing that promotes cell adhesion and proliferation, a variety of natural, synthetic, and hybrid polymers have been employed. The unique properties of electro-spun nanofiber scaffolds, such as having pores between and inside fibers and their high surface area, stimulate the reaction of fibroblast cells by rapidly activating cell signaling pathways, thereby accelerating wound healing [9, 10]. In addition, several types of hybrid nanofibers with antimicrobial effects have been developed. For instance, nanofibers containing an ethanolic extract of sour leaf incorporated with polyvinyl alcohol demonstrate antibacterial activity against *Staphylococcus aureus* [11].

Ramalingam et al. investigated the potential of Gymnema sylvestre (a plant extract) functionalized electrospun poly-ε-caprolactone nanofibers as an effective wound dressing. The presented electrospun nanofibers mat containing G. sylvestre plant extract exhibited broad-spectrum antibacterial properties and averted biofilm formation but supported human dermal fibroblasts (hDFs) attachment [12].

A nanofibrous mat of polyvinyl alcohol and chitosan (PVA/CS) containing thyme (*Thymus vulgaris*) and ginger (*Zingiber officinale*) extracts was designed by Maleki et al. using an electrospinning technique. The suggested nanofibers with an average nanoscale diameter of 382 ± 60 nm displayed high wettability, porosity and liquid absorption capacity without any adverse interaction. The proposed mat remarkably accelerated cutaneous wound healing in bacterial-infected rats by inhibiting the growth of bacteria at the wound site [13]. Cheng et al. used natural biological macromolecules, γ-polyglutamic acid and gum arabic to prepare electrospun nanofibers loaded with curcumin that had a uniform diameter, good water absorption and mechanical properties. The antimicrobial effects of suggested curcumin-loaded nanofibers against Staphylococcus aureus were investigated through an oscillating flask method. Furthermore, a mouse model with acute full-thickness skin defects was employed to investigate the wound healing capability. The results revealed that the expression of TGF-β1 and VEGF bolstered and the expression of inflammatory factors reduced, leading to an accelerated re-epithelialization process, enhanced wound contraction, and increased regeneration of new blood vessels and hair follicles [14].

Zein, a vegetable protein, has garnered considerable attention owing to its inherent traits such as biocompatibility, biodegradability, antibacterial properties, and economic viability. Consequently, it has found wide acceptance across various domains, including biomedicine, pharmaceuticals, and packaging [15]. The fibre-forming and encapsulation characteristics of zein make it a viable substitute for traditional synthetic materials. By encapsulating natural compounds within the zein nanofibers, their various properties can be improved. For instance, the high volatility and instability of the citronellol-rich *Origanum vulgare* essential oil (OEO), which has good antioxidant and antibacterial properties, limit its usage in food packaging. This problem can be solved using incorporation of OEO into electrospun zein nanofibers. According to the report of Mayire et al., the OEO-loaded zein nanofibers exhibited high thermostability, and the water contact angle was increased. Moreover, the OEO/zein nanofibers showed antimicrobial effects against *Staphylococcus aureus* and *Escherichia coli* [16].

Incorporating plants into electrospun nanofibers is a strategy to integrate the chemical and antibacterial properties of the plants and the physical characteristics of the nanofibers structure.

Since *Scrophularia striata* is a well-known plant in traditional medicine, its antibacterial, anti-inflammatory, and antioxidant properties make it a sensible adjuvant to wound care management.

As previously stated, zein, as a natural polymer, is a suitable option for wound dressing, and incorporating *Scrophularia striata* into the zein nanofibers will improve its antimicrobial qualities and wound healing potential. This can result in an effective wound dressing material with enhanced functionality compared to traditional use of *Scrophularia striata* extract in wound healing [17].

Motivated by the development of bioactive wound dressing, we design a novel zein-based nanofibrous mat containing *Scrophularia striata* plant extract.

Therefore, in this study, biocompatible zein nanofibers loaded with *Scrophularia striata extract* were produced by the electrospinning method and introduced for wound healing acceleration.

The most common characterization of the proposed nanofibrous mat has been evaluated to validate the fabrication process and biological capability. Finally, the obtained wound dressing was used to investigate in vitro and in vivo antibacterial and wound healing processes.

## Materials and methods

### Materials

*Scrophularia striata* was collected from the mountains of Ilam province, Badra city, in the spring (June) and dried at room temperature for two weeks and ground using an electric mill [15]. Zein was ordered from Sigma-Aldrich Company. Tween 80, Folin-Ciocalteau reagent and other materials used in phytochemical tests were all purchased from Merck Company.

### Preparation of *Scrophularia striata* extract

Preliminary experiments revealed that *Scrophularia striata* extracts obtained through the maceration method using ethanol (70% vol.) as solvent exhibited a more favorable antibacterial response compared to extracts obtained by Soxhlet and ultrasonic methods with ethanol (70% vol.), methanol and water as the solvents. Therefore, all subsequent investigations were conducted using the ethanol extract (70% vol.) by maceration. To prepare the mentioned extract, 10 g of dried powder from the *Scrophularia striata* plant was added to 100 mL of ethanol (70% vol.) and stirred for 72 h at room temperature. The resulting suspension was then filtered and concentrated using a rotary evaporator. The obtained extract was then subjected to freeze-drying for 24 h to achieve complete drying [18]. The appropriate amount of obtained extract was dissolved in ethanol and used as an extract solution in different experiments.

### Phytochemical tests

To identify secondary metabolites of the *Scrophularia striata* plant, several phytochemical tests were performed on the plant extract. For this purpose, 1 mL of reagents was added to 2 mL of the extract solution, with the exception of Salkowski’s test, which is used to identify terpenoids. In this test, 100 mg of dried powder was added to the reagents for Salkowski test [19].

### Preparation of zein solution

The electrospinning process was carried out on the zein solution at concentrations ranging 20–30% (w/v) with and without extract. To prepare the zein solution, an appropriate amount of zein powder was dissolved completely in ethanol 70% vol., under stirring for 2 h. Then, in order to decrease the viscosity and surface tension of the solution, 121 mg of tween 80 as a surfactant was added to 5 mL of solution and stirring was continued until a homogeneous zein solution was obtained [20]. To prepare the zein solution containing the extract, 1 mL of the ethanolic maceration extract solution of the *Scrophularia striata* plant with a concentration of 400 mg mL^−1^ was added to 5 mL of the zein solution and then it was stirred for three hours at room temperature. In order to obtain high-quality electrospun fibers, the amounts of materials used in the preparation of the solution were optimized.

### Electrospinning process

The zein solution (5 mL) was transferred to a syringe with a number 18 needle head and placed in the electrospinning instrument. Syringe pump was used to feed the polymer solution to the needle tip at a feeding rate of 1 mL h ^−1^. To facilitate the collection of nanofibers post-electrospinning, an aluminum foil was placed on the collector, vertically 10 cm away from the needle tip (TCD = 10 cm) and the rotating plate was set to 200 rpm. Electrospinning process was carried out at room temperature and under a constant electric field of 14 kV [21].

### Scanning electron microscopy (SEM)

The morphology of electro-spun nanofibers was evaluated by a SEM device (LEO 1450 VP model, made in Germany). Digimizer image visualization software was used to calculate the nanofiber diameter. To draw the histogram showing the size distribution of electro-spun nanofibers, about 50 random fibers were selected from SEM images, and the average data was considered as the average diameter of the fibers.

### Energy dispersive X-ray (EDX) analysis

EDX and mapping analysis were performed to identify atoms and investigate the distribution of elements in zein nanofibers containing extract and without extract by the EDX device (TE scan-mira3, Czech Republic).

### Fourier transform infrared (FT-IR)

The FT-IR spectra were recorded using a PerkinElmer (Spectrum Two, USA) spectrometer to investigate the functional groups of the materials in the samples, including extract, zein nanofibers, and nanofibers loaded with extract. All spectra were recorded in the range of 400–4000 cm^−1^.

### Contact angle analysis

To determine the amount of hydrophilicity and hydrophobicity of zein nanofibers with and without extract, an angle contact test was performed (Ramé-Hart, USA). In this way, 1 × 1 cm pieces of nanofibers were cut using a contact angle protractor, accompanied by a high-quality and high-speed digital camera (1 min, 25 fps at 25 ˚C) to measure the contact angle between the drop of deionized water and the nanofibers.

### Antibacterial activity

To evaluate the antibacterial properties of the hydro-ethanolic (ethanol 70%) maceration extract of the *Scrophularia striata* and the electro-spun nanofibers containing the extract, antibacterial power tests were performed.

### Antibacterial activity of the hydro-ethanolic extract by disk diffusion method

Four different concentrations of the maceration extract prepared. For this purpose, 100, 200, 300 and 400 mg of dried extract were solved in 1 mL^−1^ of ethanol 70% vol. Sterile blank discs (6.4 mm) were then put inside the microtubes to be saturated with the extract. After 20 min, the discs were taken out and placed in sterile petri dish to reduce humidity and then used in Kirby-Bauer disc diffusion assay for finding antibacterial activity. For this purpose, *Staphylococcus aureus* (ATCC 6538), *Bacillus subtilis (*ATCC 6633), *Escherichia coli* (ATCC 25922) *and Pseudomonas aeruginosa* (ATCC 9027) were inoculated in Mueller-Hinton broth and incubated at 37 °C till its turbidity be equal to 0.5 McFarland turbidimeter. Subsequently a lawn culture was prepared on Mueller-Hinton agar using sterile swabs and the prepared discs were put on the culture surface. The cultures were incubated at 37 °C for 24 h and the diameter of the growth inhibition zone around the disc was measured and recorded (mm) [22].

### Minimum inhibitory concentration (MIC) and minimum bactericidal concentration (MBC)

In order to find the least concentration that can inhibit bacterial growth or kill them, these tests were applied. Two-fold serial dilutions from the extract, i.e., 100, 50, 25, 12.5 and 6.25 mg mL^−1^ were prepared in Mueller- Hinton broth and 0.1 mL of bacterial fresh culture with 0.5 McFarland turbidity was inoculated and incubated (37 ˚C for 24 h). The bacterial growth was confirmed based on turbidity of culture medium and those tubes remained clear were regarded as growth negative. The least concentration that inhibited bacterial growth was regarded as MIC. Subsequently, a loopful from growth negative tubes was cultured on the surface of Mueller-Hinton agar and incubated (37 ˚C for 24 h). The least concentration that inhibited colony formation was regarded as MBC [23, 24].

### Antibacterial activity assessment of nanofibers

The antibacterial activity of synthesized nanofibers was evaluated by three different methods. In the first method, the standard Kirby-Bauer disc diffusion method was followed. Two pieces (1 × 1 cm) of zein nanofibers containing extract were prepared and only one of them was exposed to UV radiation for 15 min at a distance of 30 cm before being placed in the culture medium to minimize environmental contamination. Then, both irradiated and non-irradiated samples were then placed on the lawn culture prepared as described above and incubated at 37 °C for 24 h. The clear halo zone that reveals bacterial growth inhibition was measured and recorded (mm) [25].

In the second method the antibacterial potential of zein nanofibers was evaluated in the broth culture medium. Pieces of zein nanofibers (3 × 3 cm) containing extract were prepared and irradiated under UV light for 15 min at a distance of 30 cm. After that, each piece was transferred to 3 mL of Mueller-Hinton broth culture medium inoculated with 300 µl of fresh culture of bacteria with 0.5 McFarland turbidity and incubated (37 °C, 24 h). For each bacterium a culture without zein nanofiber was prepared and regarded as control. Following incubation, the absorbance (600 nm) of the treated and control samples was measured. In case of bacterial growth inhibition, the absorbance of treated bacteria will be less than control [26].

In the third method the antibacterial activity of zein nanofibers was investigated through colony count of treated bacteria. Pieces (3 × 3 cm) of zein nanofibers containing extract were irradiated by UV light at a distance of 30 cm for 30 min. Then, each piece was inserted in to Erlenmeyer’s flasks containing10 mL of Mueller-Hinton broth and 100 µL of 0.5 McFarland suspension of target bacterium and then incubated (37 °C, 30 rpm and 24 h). Similarly, controls were regarded without zein nanofibers. After that, serial tenfold dilutions till 10^−6^ were prepared and duplicate streak cultures were prepared from them on Nutrient agar. Following incubation (37 °C, 24 h) the number of colonies was counted and reported as CFU mL^−1^. In case of antibacterial activity, the total number of bacteria exposed to zein nanofibers will be reduced in comparison with control [27].

### Total biophenol content release assessment

#### Poly phenolic compounds assay of extract

The total content of biophenolic compounds in the extract of *Scrophularia striata* was evaluated based on the Folin-Ciocalteu reagent [28]. For this purpose, 0.1 g of ethanolic maceration extract was dissolved in 8 mL of water by stirring. After centrifugation, the clear solution was diluted to a volume of 10 mL in a volumetric flask. In the next step, 0.1 mL of this solution was transferred to a 10 mL volumetric flask, followed by the addition of 0.3 mL of Folin reagent and 1.5 mL of sodium carbonate solution (20% w/v), and then diluted up to 10 mL. Finally, the absorbance of the solution was recorded at 760 nm after one hour using a Jenway UV-Vis spectrophotometer (model 6715, UK). In order to draw the calibration curve, gallic acid was used as a standard and the same procedure was also run on the different concentrations of gallic acid [29].

#### Biophenol content release from electrospun nanofibers

A piece of 3 × 3 cm^2^ dimensions (the same size used for antibacterial activity studies) was prepared from zein nanofibers without extract and zein nanofibers containing extract. Each piece was divided into 9 pieces of 1 × 1 cm^2^ and placed in 5 mL of phosphate-buffered saline (PBS) solution (pH = 7.4). Then, the release of bio-phenol content in both nanofibers was checked during 24 h at specific time intervals. For this purpose, at predetermined intervals, 0.2 mL of each solution was sampled, and Folin’s test was conducted according to the standard protocol to calculate the total bio-phenol content. To maintain a constant total volume, after each sampling, an equivalent volume of fresh PBS solution was replaced [30].

### Biocompatibility and biodegradability

#### Toxicity of extract on cells by MTT method

Fibroblast cells (10,000 cells per well) were seeded into the wells of a 96-well plate containing DMEM/F12 medium and incubated for 24 h. The medium of the well was then discarded and 100 µL of the medium containing the maceration extract of the plant at concentrations of 0.01, 0.1, 1, 10 and 50 mg mL^−1^ was added to the well. Again, after another 24 h of incubation, the contents of the wells were discarded and approximately 100 µL of MTT solution with a concentration of 0.5 mg mL^−1^ was added to each well. Following 4 h of incubation, the MTT solution was removed from the wells and 100 µL of DMSO solvent was added to the wells to dissolve the purple formazan crystals. After 30 min, the absorbance of the solutions was read at a wavelength of 570 nm [31, 32].

#### Evaluation of cell survival on nanofiber by MTT method

For this test, nanofibers with a diameter of 6 mm were prepared using a punch. The nanofibers were then exposed to ultraviolet radiation for 2 h for sterilization. The desired nanofibers were placed in the wells of a 96-well plate and 3% antibiotic was placed on them for 24 h. Then, 10,000 fibroblast cells were cultured on zein nanofibers without extract and zein nanofibers containing extract in two time periods of 24 h and 48 h, after which the MTT test was performed [33].

#### In vitro and in vivo studies

To investigate the biocompatibility and biodegradability of nanofibers containing extracts, the implantation method was employed using animal models. To this end, pieces with a diameter of 12 mm were prepared from zein nanofibers without extract and zein nanofibers containing extract. These samples were then implanted under the skin of two groups of BALB/c mice for a period of two and four weeks. Nanofibers without extract were implanted in the neck area of each mouse, while nanofibers containing extract were implanted in the back area. Before initiating the procedure, the rats were anesthetized with a mixture of ketamine in a concentration of 160 mg kg^−1^ and xylazine in a concentration of 4 mg kg^−1^. After two and four weeks, each group of mice was euthanized easily, and the skin containing the nanofibers was dissected. The samples were fixed in 10% formalin for 48 h [34]. Subsequently, histological analysis was performed using paraffin embedding, standard histotechnical procedures, and H&E staining [35].

All procedures were evaluated and approved by the Shahid Chamran University Local Ethic Committee for Animal Experiments (EE/1401.2.24.225871/scu.ac.ir).

### Statistical analysis

This study reports all values as means ± standard deviation (SD), with each measurement performed in triplicate. Statistical analysis was carried out using one-way ANOVA, followed by unpaired Tukey’s test, and a *p*-value of less than 0.05 was deemed statistically significant.

## Results and discussion

### Secondary metabolites

Secondary metabolites of plants such as alkaloids, flavonoids, glycosides, tannins, saponins and terpenoids have been widely used as valuable compounds in several industries namely pharmaceuticals, cosmetics, food, etc. It is obvious that these compounds dramatically increase the importance and commercial value of plants [36]. Table 1 shows the secondary metabolites of the *Scrophularia striata* plant, resulting from phytochemical tests performed on the solution of the maceration extract. According to the results, this extract did not contain only glycosides. The presence of secondary metabolites in *Scrophularia striata* makes the extract of this plant have antimicrobial activity against microorganisms [36].

**Table 1:** Obtained results from phytochemical tests on maceration extract of the *Scrophularia striata* plant

Active ingredient	Test type	Sign	Response ^a^
Alkaloid	Dragendorff’s reagent	Sediment or orange color	+
	Wagner’s Reagent	Sediment or red-brown color	+
Flavonoids	Base reagent	Yellow or red	+
Glycoside	Fehling’s solution	Brick red sediment	-
Tannin	Ferric Chloride.	Green-brown or blue-black color	+
Saponin	Foaming	Production of foam	+
Terpenoid	Salkowski reagent	The formation of a red-brown layer on the surface	+

a: + Positive response, - Negative response

### Optimization of parameters affected on nanofibers morphology

**Fig. 1:**
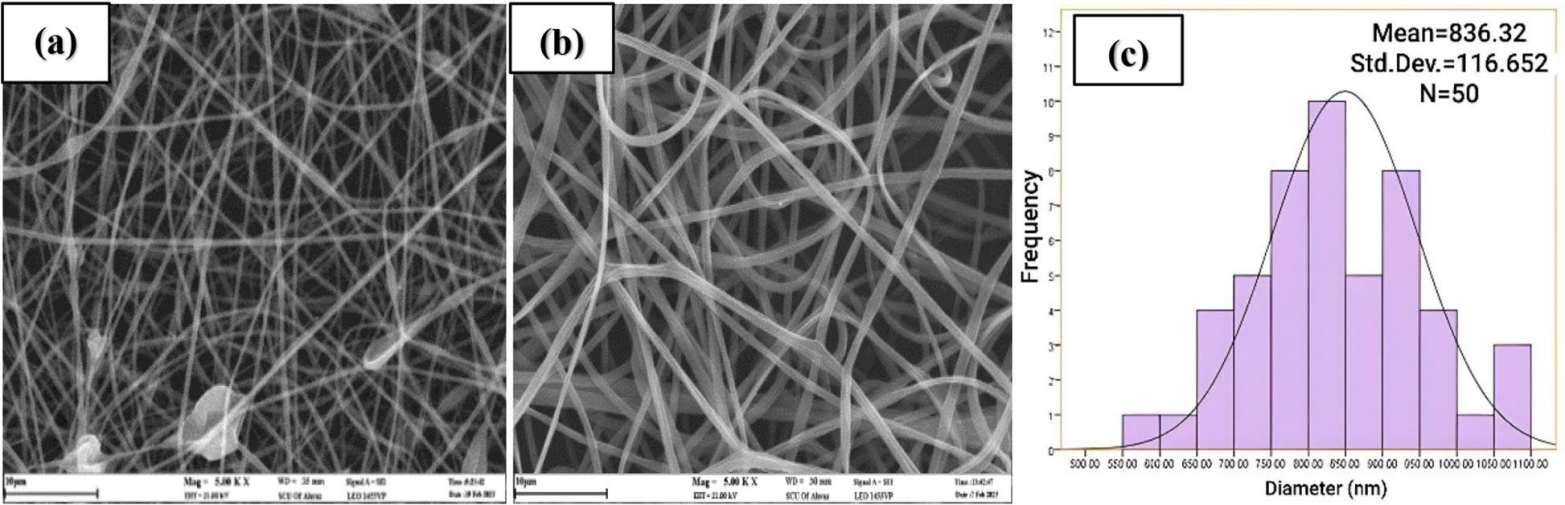
SEM images of electrospun zein nanofibers in concentrations (**a**) 20% (w/v), (**b**) 25% (w/v), and (**c**) image of histogram of diameter size distribution of electrospun zein nanofibers with 25% (w/v) concentration. [Image scale: 10 μm, solution volume: 5mL, amount of tween 80: 121 mg, solvent: ethanol 70% vol., TCD: 10 cm, drum speed = 200 rpm, applied voltage : 14 kV, feeding rate: 1 mL h ^−1^, temperature : 25 °C ]

In order to optimize effective parameters for obtaining a high-quality scaffold, the morphology of prepared electrospun nanofibers in different conditions was investigated by SEM images. The viscosity is one of the most influential factors in the morphology of nanofibers [37]. Therefore, the morphology of nanofibers was studied in different concentrations of zein dissolved in ethanol 70% vol. containing tween 80, while having different viscosities. According to Fig. 1a, the formed nanofibers at a low concentration of zein (20% w/v) have many beads. Because the viscosity of the polymer solution is low, the surface tension of the solution causes the polymer chains to break into pieces before reaching the collector. This event causes beads to form between the nanofibers [38]. By increasing the concentration to 25% (w/v), uniform nanofibers without beads with an average diameter of 836.3 nm are formed (Fig. 1b). When the viscosity of the solution is high, the degree of entanglement of the polymer chains increases, they can overcome the surface tension and cause the formation of beadless nanofibers [39]. Also, the histogram of the diameter size distribution of nanofibers at a zein concentration of 25% (w/v) is shown in Fig. 1c. However, increasing the concentration to 30 and 35% (w/v) during the electrospinning process leads to the formation of droplets due to the interruption of continuous fluid flow, attributed to the blockage of the flow of the solution at the tip of the needle [40]. Therefore, a concentration of 25% (w/v) was selected as the optimal amount of zein.

**Fig. 2:**
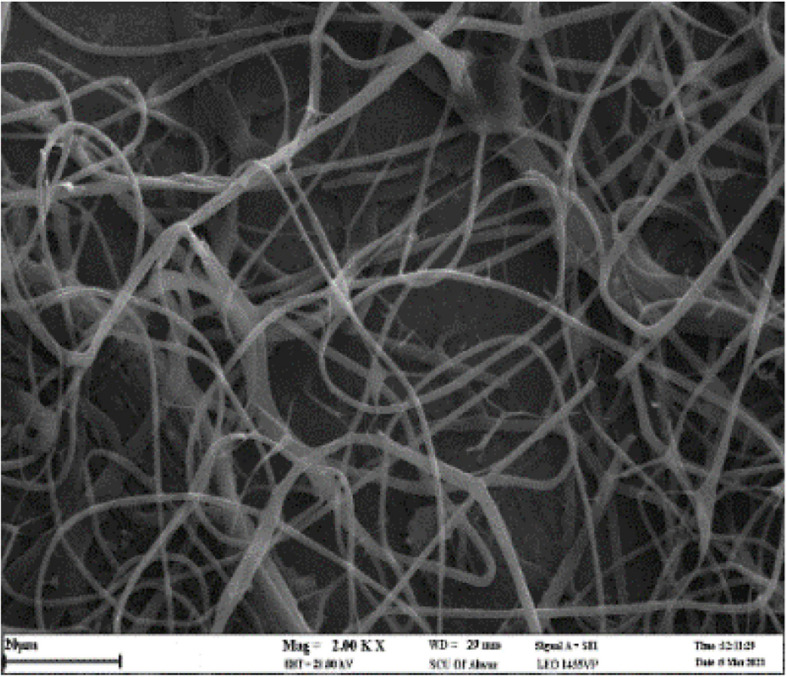
SEM image of zein nanofibers prepared in 60% vol. ethanol [Image scale: 20 μm, solution volume: 5mL, amount of tween 80: 121 mg, zein concentration: 25% (w/v), TCD: 10 cm, drum speed = 200 rpm, applied voltage: 14 kV, feeding rate: 1 mL h ^−1^, temperature: 25 °C]

The volume percentage of ethanol as the solvent used in the preparation of the zein solution plays a significant role in the electrospinning process [41]. In order to study this parameter, the zein solutions (25% w/v) were prepared by dissolving an appropriate amount of zein in ethanol with different volume percentages of 50, 60, 70 and 80%, and then the electrospinning process was performed on them. The 50% vol. ethanol was unable to dissolve zein, making it unsuitable for electrospinning. Furthermore, the SEM image presented in Fig. 2 illustrates that using 60% vol. ethanol resulted in the formation of non-uniform nanofibers. Conversely, employing 70% vol. ethanol (Fig. 1b) yielded uniform nanofibers, whereas the use of 80% vol. ethanol produced nanofibers lacking sufficient consistency to separate from the aluminum foil surface. Therefore, 70% vol. ethanol was chosen as the optimal solvent.

As mentioned earlier, tween 80 as a surfactant was used in order to reduce the viscosity and surface tension of the solution [20]. Therefore the effect of the amount of tween 80 added to 5 mL of zein solution for electrospinning was investigated. The obtained results indicated that by increasing the amount of tween up to 121 mg, the viscosity was reduced and the electrospinning process was performed more easily at this value. However, by adding more of this surfactant to the zein solution, the solution became sticky and prevented the process from continuing, so the amount of 121 mg of tween 80 was considered as optimal.

**Fig. 3:**
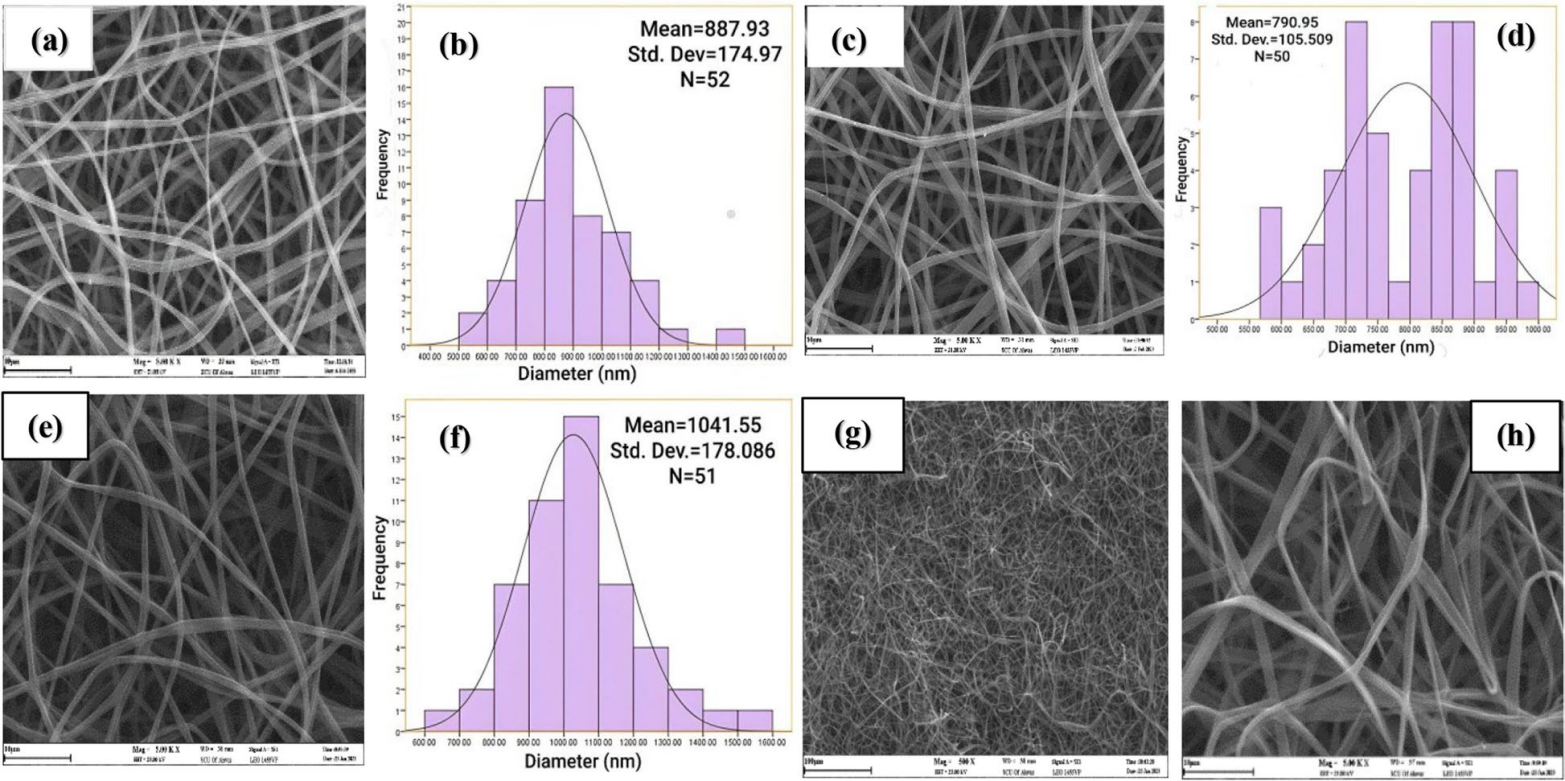
SEM image and histogram of diameter size distribution of nanofibers at voltage (**a** and **b**) 12 kV, (**c** and **d**) 14 kV, (**e** and **f**) 17 kV; SEM images at (**g**) 20 kV and (**h**) 23 kV. [Image scale: 10 μm, zein concentration: 25% (w/v), solution volume: 5 mL, amount of tween 80: 121 mg, solvent: ethanol 70% vol., TCD: 10 cm, drum speed = 200 rpm, feeding rate: 1 mL h ^−1^, temperature: 25 °C]

**Fig. 4:**
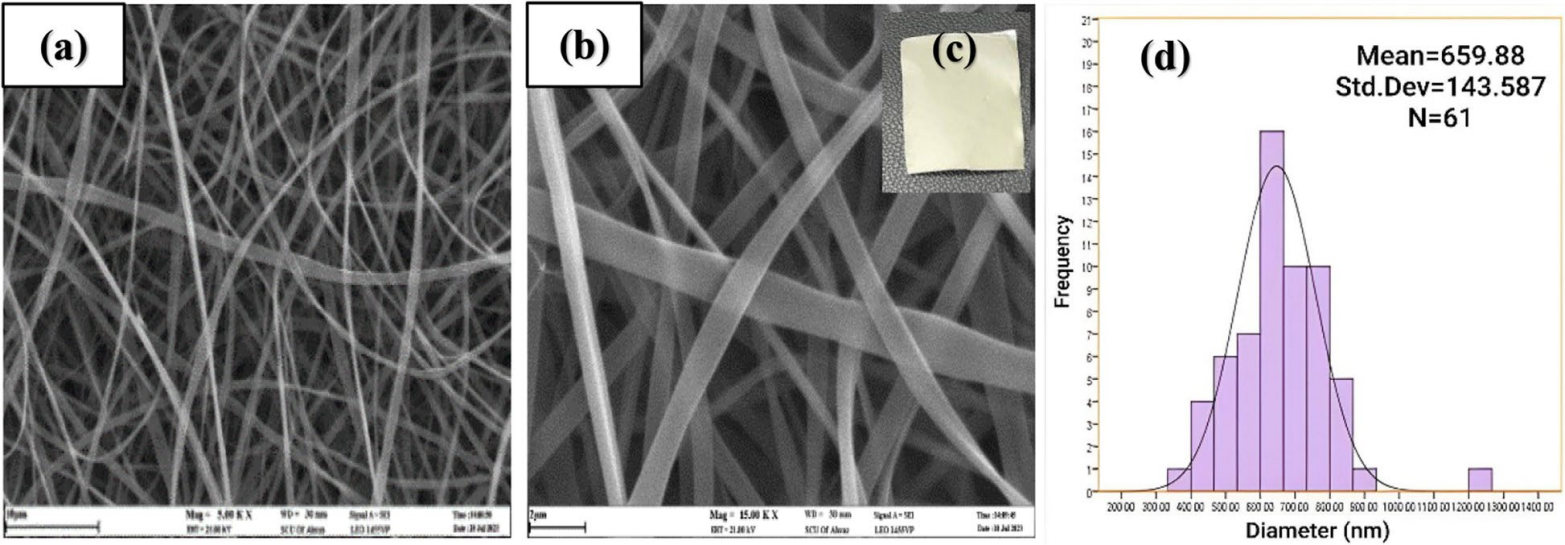
SEM images of zein nanofibers containing the maceration extract of the *Scrophularia striata* plant (**a**) at the scale of 10 μm, (**b**) at the scale of 2 μm, (**c**) picture of zein nanofibers, and (**d**) histogram of the diameter size distribution of nanofibers containing the extract. [Zein concentration: 25% (w/v), solution volume 5mL, amount of tween 80: 121 mg, solvent: ethanol 70% vol., amount of extract: 6.7% (w/v), TCD: 10 cm Drum speed = 200 rpm, applied voltage = 14 kV, Feeding rate: 1 mL h ^−1^, Temperature: 25 °C]

**Fig. 5:**
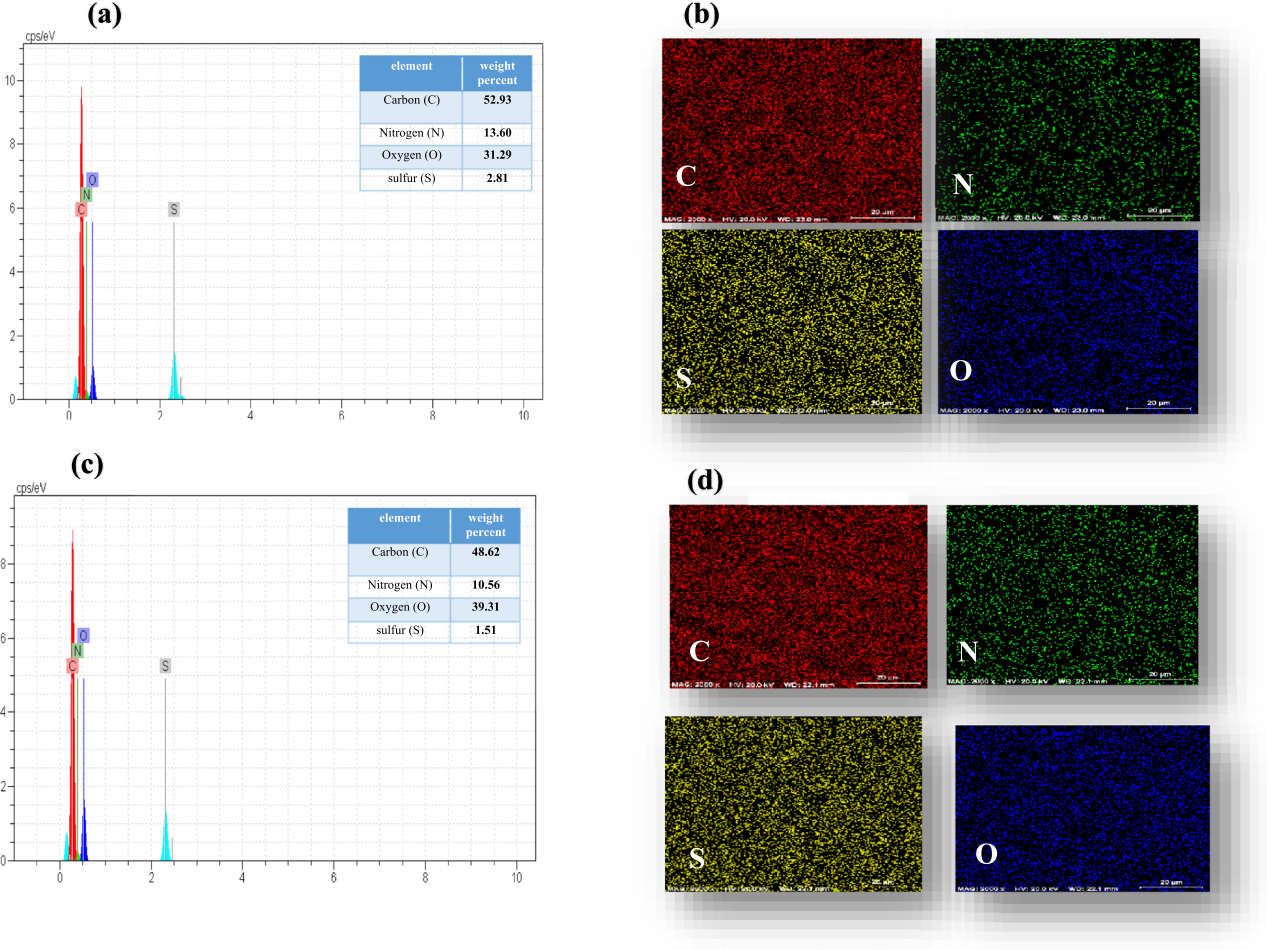
EDX analysis and elemental mapping of zein nanofibers (**a**, **b**) without extract; (**c**, **d**) containing the extract of the Scrophularia striata plant

**Fig. 6:**
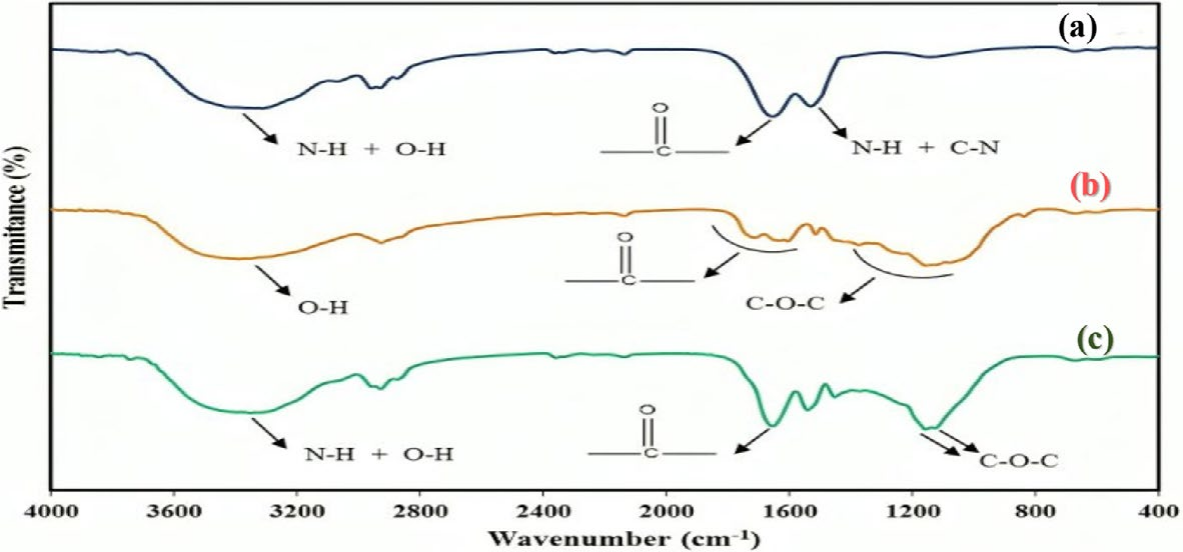
FT-IR spectrum; (**a**) zein nanofibers, (**b**) *Scrophularia striata* extract prepared by the maceration method, (**c**) zein nanofibers containing the *Scrophularia striata* extract

**Fig. 7:**
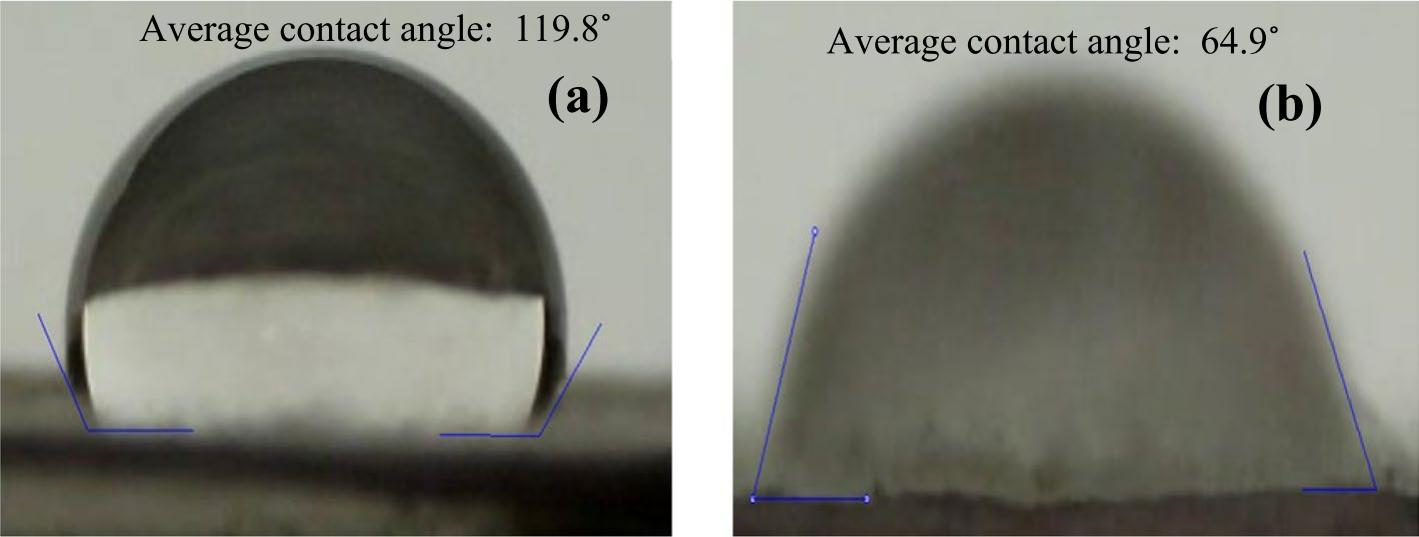
Images of the water contact angle and its value for zein nanofibers (**a**) without extract, (**b**) containing the *Scrophularia striata* extract

**Fig. 8:**
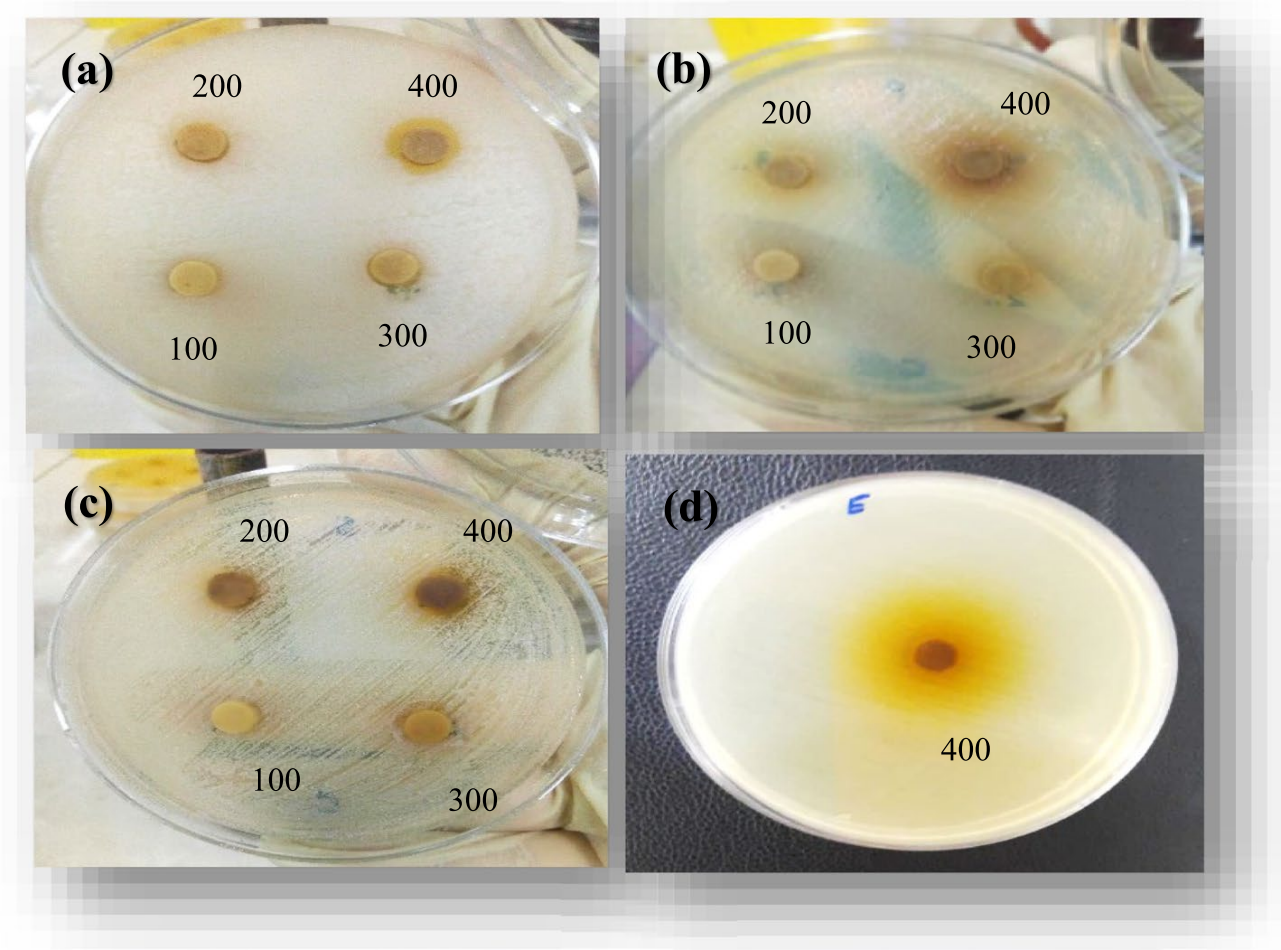
The growth inhibition halo zone of the maceration extract at 100 to 400 mg mL^−1^ concentrations against (**a**) *Bacillus subtilis*, (**b**) *Pseudomonas aeruginosa*, (**c**) *Staphylococcus* aureus (the numbers written in each figure indicate the concentration of the extract), and (**d**) growth inhibition halo zone of the maceration extract at of 400 mg mL^−1^ a concentration against *Escherichia coli*

**Fig. 9:**
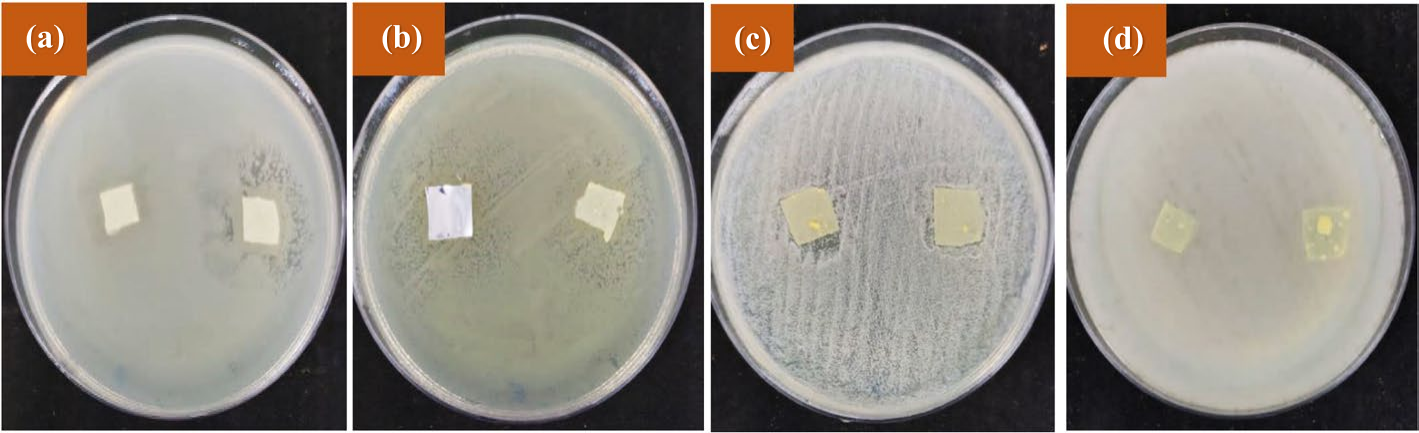
The halo of growth inhibition of zein nanofibers containing the maceration extract of the *Scrophularia striata* against **a*** Pseudomonas aeruginosa*, **b ***Escherichia coli*, **c*** Staphylococcus aureus*, **d*** Bacillus subtilis.* (In each plate, the nanofibers on the right side were exposed to UV radiation and the nanofibers on the left side were not exposed to radiation)

**Fig. 10:**
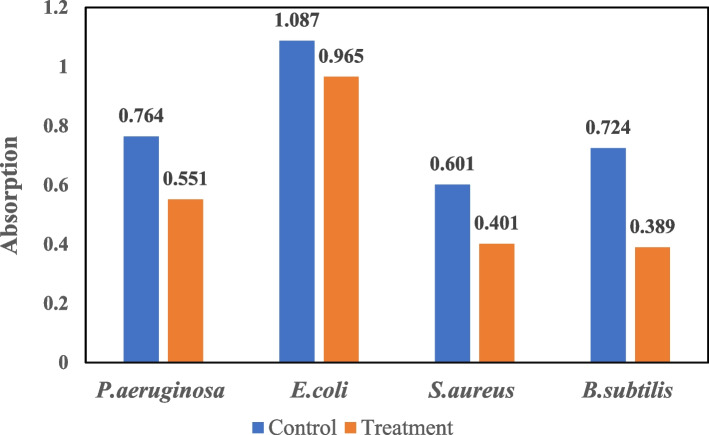
The results of bacterial growth in the absence (control) and presence of zein nanofibers containing maceration extracts. The numbers show bacterial suspension absorbance in 600 nm

**Fig. 11:**
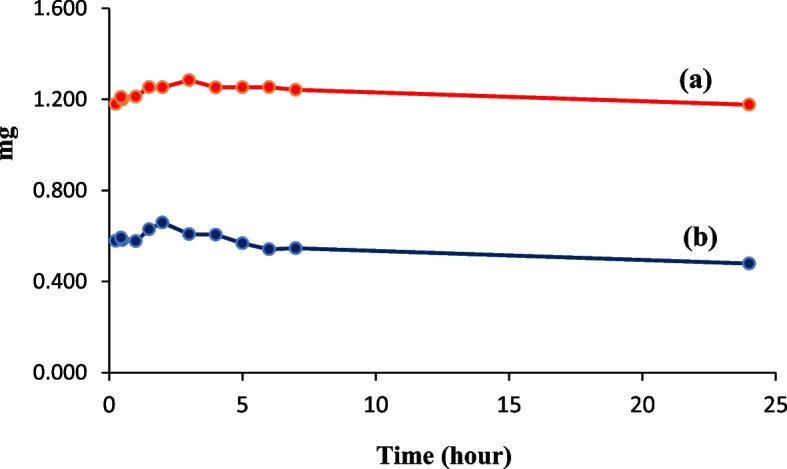
Biophenol release rate during 24 h for zein nanofibers containing the maceration extract of *Scrophularia striata* at room temperature in (**a**) PBS solution and (**b**) water

**Fig. 12:**
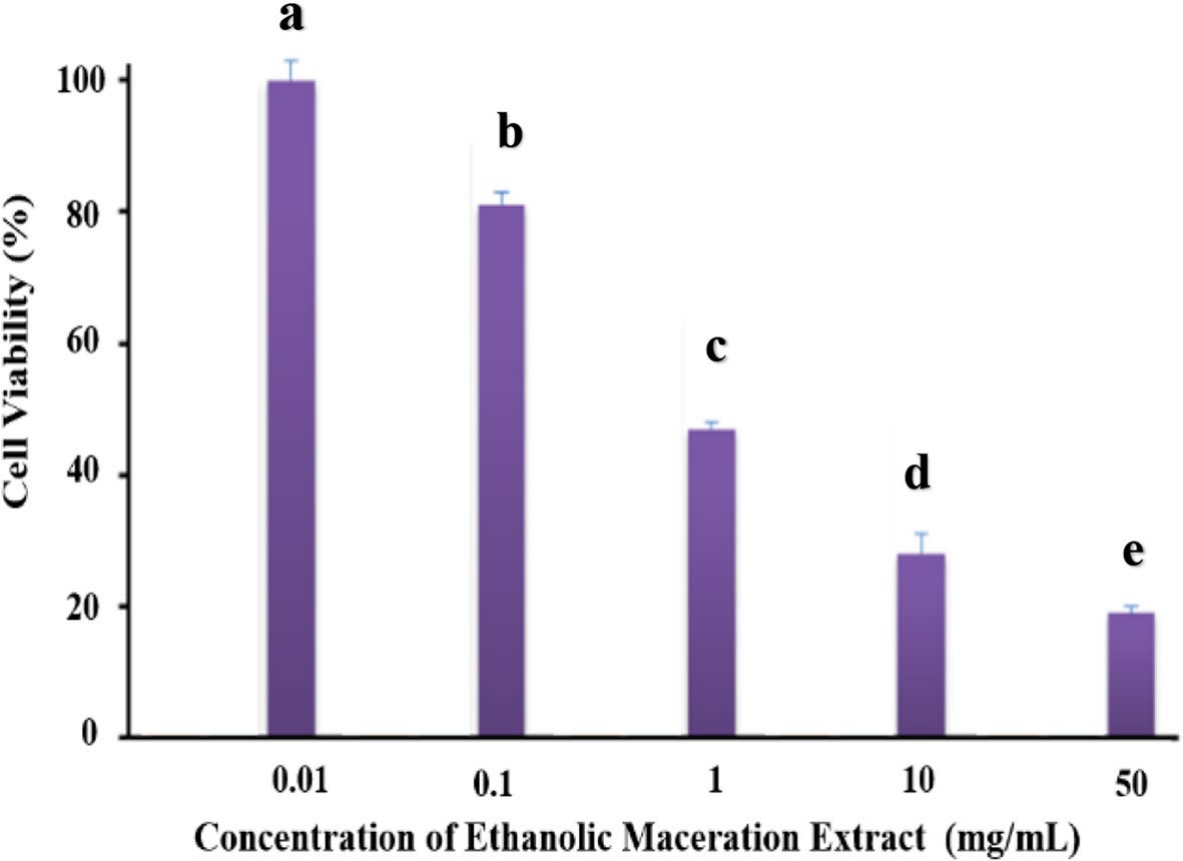
Investigating the percentage of survival of fibroblast cells treated with different concentrations of maceration extract of the *Scrophularia Striata* plant using the MTT test after 24 h. Dissimilar letters indicate significance of *P* < 0.05

**Fig. 13:**
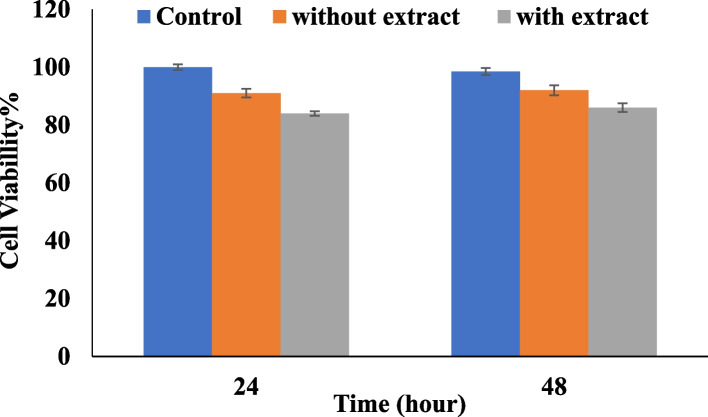
Percentage of cell survival using MTT test for zein nanofibers without extract and zein nanofibers containing extract on fibroblast cells after 24 and 48 h. Fibroblast cells cultured in 2D were used as a control sample

**Fig. 14:**
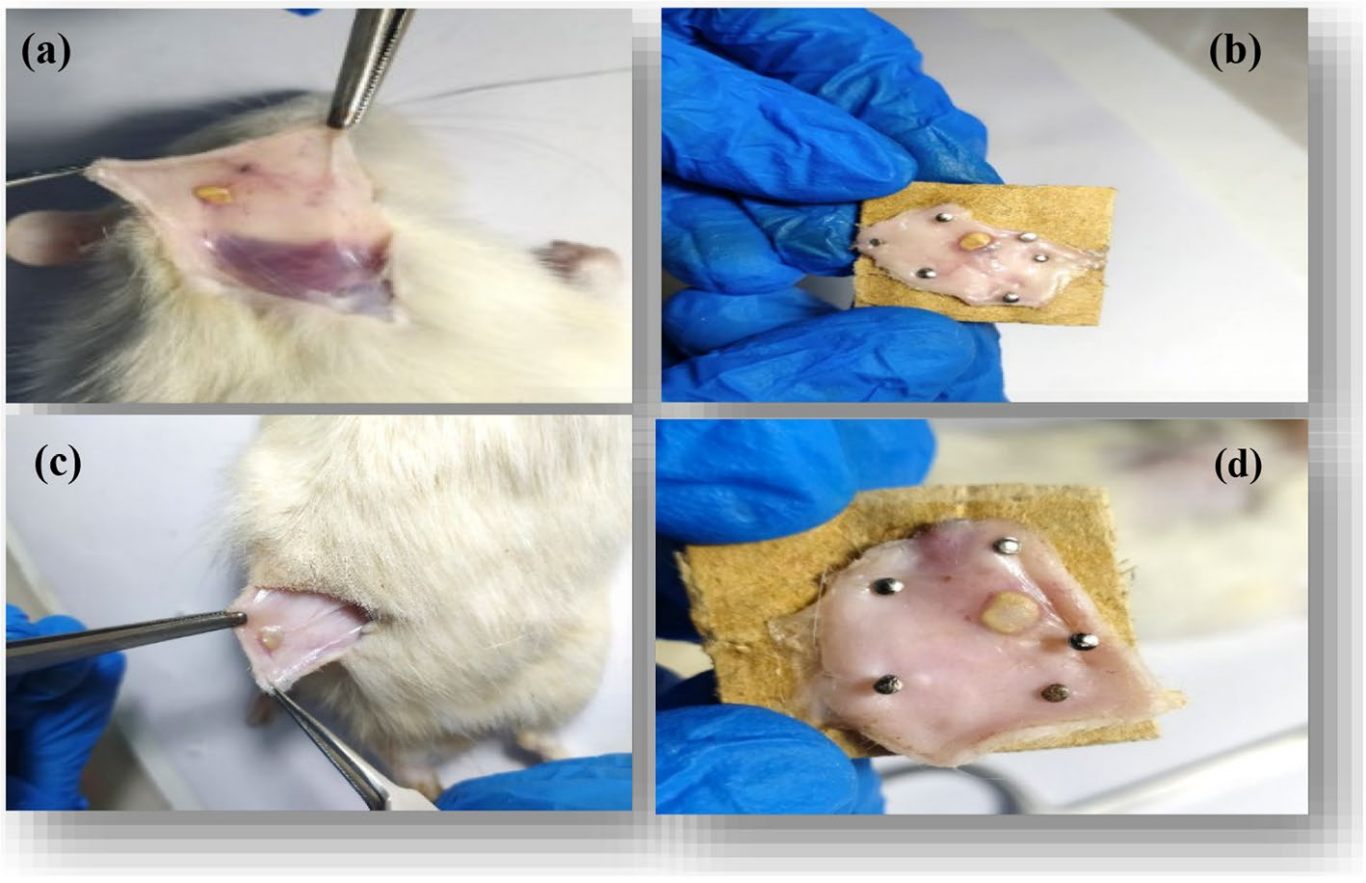
**a** Presence of residues from the nanofibers without extract after one month in the neck area of mice. **b** Preparation of skin tissue isolated from the neck area along with nanofibers without extract for histomorphology tests. **c** Presence of residues from the nanofibers with extract after one month in the back area of mice. **d** Preparation of skin tissue isolated from the back region for histomorphology tests

Figure 3 shows pictures of nanofibers made of zein by the electrospinning process at different voltages of 12, 14, 17, 20, and 23 kV. Increasing the voltage from 12 kV (Fig. 3a) to 14 kV (Fig. 3c) resulted in a reduction in the average fiber diameter, but beyond 14 kV, the average diameter started to increase again, and it peaked at 17 kV (Fig. 3e). However, with a further increase in voltage (20 kV and 23 kV values), nanofibers with inappropriate morphology and significant accumulation were formed (Fig. 3g and h). The reason behind this can be attributed to the exit of more polymer solution from the tip of the needle, which causes the flight time of the jet to decrease and to collect faster on the collector [42]. At 12 kV, the nanofibers have a larger average diameter (Fig. 3b) due to a slight increase in solution viscosity, which hinders jet formation and dispersion during the electrospinning process. In contrast, at 17 kV, there is a significant increase in the average amount of nanofiber diameter (Fig. 3f). Since uniform nanofibers with an average diameter of 790.95 ± 105.509 nm (*n* = 50) were obtained at a voltage of 14 kV (Fig. 3a), it was chosen as the optimal voltage in the electrospinning process.

Subsequently, zein nanofibers containing the extract were prepared under optimum conditions by adding the ethanolic maceration extract solution of the *Scrophularia striata* plant. The uniform structure of the nanofibers and their average diameter of 659.8 ± 143.5 nm (*n* = 50) were confirmed by the SEM images displayed in Fig. 4, which also reveal a decrease in nanofibers diameter after adding the extract. The reduction in viscosity observed after adding the extract to the zein solution can be attributed to several factors. One of these factors is that bioactive compounds and surfactants or surface-active compounds in plant extracts can reduce viscosity and surface tension of the solution during the process, resulting in a reduced fiber diameter. Additionally, electrostatic interactions between the extract and polymer solution can lead to a more uniform distribution of charge across the solution, facilitating the formation of fibers with a smaller diameter [43–45]. Similar results have been reported for *Sorghum* and *Poria cocos* extract [20].

### Characterization of nanofibers

The presence of elements in zein nanofibers was evaluated by EDX analysis. Figure 5a illustrates the percentage of elements present in zein nanofibers without extract. The presence of sulfur is due to cysteine, which shows that there is a small amount of *β*- and *γ*-zein along with *α*-zein [46]. The EDX mapping of elements shown in Fig. 5b affirms uniform distribution of elements in the nanofibrous structure. Furthermore, the EDX spectrum and elemental mapping of zein nanofibers containing the maceration extract of the *Scrophularia striata* plant are shown in Fig. 5b and c.

FT-IR spectra were used to investigate the presence of *Scrophularia striata* extract in the zein nanofibers. In Fig. 6a, the spectrum of zein nanofibers without extract displays characteristic peaks appearing at 1652 cm^−1^ and 1525 cm^−1^, attributed to the first and second type amide stretching bands, respectively. The first type peak amide is related to the stretching vibration bands of the carbonyl group (C = O) and the second type peak amide is related to N-H bending vibrations coupled with C-N stretching vibrations. The peak at 3350 cm^−1^ is related to the overlap of stretching vibration of O-H and N-H groups [47]. Figure 6b shows the spectrum of the ethanolic maceration extract of the plant. The main peaks in the range of 1372 –1072 cm^−1^ correspond to the C-O-C stretching vibration of the ether group, while the peaks in the range of 1707 –1606 cm^−1^ and 13,359–13,359 cm^−1^ represent the stretching vibrations of the carbonyl group (C = O) and the phenolic group (O-H), respectively [48]. In the spectrum of zein nanofibers containing extract (Fig. 6c), peaks corresponding to both zein and the zein extract are observed. The peaks in the region of 1159 cm^−1^ and 1126 cm^−1^ are related to the C-O-C group of the components presence in plant extract, while peaks at 1650 cm^−1^ are attributed to the overlapping carbonyl groups of the extract and zein. The peak in the area of 3348 cm^−1^ results from the overlap between hydroxyl and amine groups [49]. These observations collectively confirm the presence of *Scrophularia striata* extract within the zein nanofibers.

### Water contact angle measurements

The wound site secretes a variety of substances in addition to dead tissue and bacteria. Eliminating superfluous secretions speeds up the healing process and helps avoid infection. Thus, in order to guarantee tissue hydration and preserve the extracellular space, it is essential to assess the degree of wettability of designed nanofibers [50]. The hydrophobicity of the nanofibers is proportional to the contact angle between the water drop and the nanofibers. A larger contact angle between the water drop and the nanofibers indicates that the nanofibers are more hydrophobic, and a smaller contact angle reveals a more hydrophilic nanofibers [51]. Figure 7a and b display the average contact angle between water drop and nanofibers without and with *Scrophularia striata* extract, which are 119.8° and 64.9°, respectively. This means that nanofibers containing extract have more hydrophilic properties than nanofibers without extract. Hydrophilicity is an important factor that can increase the efficiency of wound healing. Hydrophilic nanofibers can quickly absorb wound secretions and keep the wound environment away from contamination [52]. Moreover, the hydrophilicity of nanofibers helps to keep the wound wet and prevents drying and crust development [53].

### Antibacterial activity

#### Antibacterial activity of extract by disc diffusion method

The inhibition zone (halo diameter) around each disc was measured (mm) in three directions and the average was reported in Table 2 and shown in Fig. 8. A larger halo diameter indicates a greater ability of the extract to inhibit bacterial growth [54]. In case of complete growth inhibition, it was reported as bactericidal while if growth retardation happened, it was reported as bacteriostatic. It is worth mentioning that when the concentration of the ethanolic maceration of the plant is 400 (mg mL^−1^), it has the highest Bacteriostatic and Bactericidal abilities, especially against gram-positive bacteria.

The MIC and MBC indices were investigated in order to find the least concentrations at which the bacterial growth is inhibited or bacterial death will happen, respectively [55]. The results are shown in Table 3. According to these results, ethanolic maceration extract showed bacteriostatic effect against four tested bacteria, and lowest MIC was found against Gram-positive bacteria, i.e., *B. subtilis and S. aureus* while the *B. subtilis* was more sensitive to this extract than S. aureus and this bacterium was inhibited in lower concentration of the extract. So, this extract can have both bacteriostatic and bactericidal effects against Gram-positive bacteria depending on its concentration [56]. While in case of Gram-negative bacteria the MIC was equal to MBC against *P. aeruginosa* and *E. coli* and this extract showed a bactericidal effect against these types of bacteria. Furthermore, the highest MIC and MBC indices were found for *P. aeruginosa* that show resistance of this bacterium to the antibacterial compounds in this extract at low concentration while it can be inhibited at much higher concentrations. The lower sensitivity of Gram-negative bacteria to this extract may be due to the presence of an outer membrane that limits access of active compounds to their targets. Bacterial death can be as a result of destruction and leakage of cell contents of macromolecular structures like as outer membrane, cell wall and cytoplasmic membrane or due to damage to polymeric substances in the bacterial cell including proteins and nucleic acids [57].

**Table 2:** Halo diameter measured in the disc diffusion test for discs impregnated with extracts of *Scrophularia striata* plant with different concentrations

Concentration of extract (mg mL −1 )	Halo diameter (mm)							
	*Bacillus subtilis*		*Staphylococcus aureus*		*Pseudomonas aeruginosa*		*Escherichia coli*	
	Bacteriostatic	Bactericidal	Bacteriostatic	Bactericidal	Bacteriostatic	Bactericidal	Bacteriostatic	Bactericidal
100	8	-	9	-	-	-	-	-
200	9	-	9	-	-	-	-	-
300	^_^	10	10	-	8	-	-	-
400	12	10	12	8	9	-	-	9

**Table 3:** The results of the (MIC) and (MBC) for the maceration extract

Bacterial species	Minimum Inhibitory Concentration (MIC, mg mL^−1^)	Minimum Bacteriocidal Concentration (MBC, mg mL^−1^)
*Bacillus subtilis*	6.25	25
*Staphylococcus aureus*	12.5	25
*Pseudomonas aeruginosa*	50	50
*Escherichia coli*	25	25

The antibacterial activity of this extract is due to the bioactive compounds present in this plant. Five known compounds, including cinnamic acid, three flavonoids (quercetine, isorhamnetin-3-O-rutinoside and nepitrin) and one phenyl propanoid glycoside (acteoside 1) have been isolated from *S. striata* Boiss [58]. Bagheri et al., also investigated the antibacterial activity of *Scrophularia striata* and stated that phenolic compounds present in this plant is responsible for antibacterial activity [59]. The hydroxyl group of the phenolic ring forms hydrogen bonds and also dissipates the proton gradient over the bacterial cytoplasmic membrane. It causes changes in proton motive force (PMF), depletion of intracellular ATP pool and finally leads to impairment of essential functions of the bacterial cell [60]. Different mechanisms are responsible for antibacterial activity of flavonoids. They inhibit nucleic acid synthesis, functions of cytoplasmic membrane, energy metabolism, attachment and biofilm formation by bacteria, porin function on the cell membrane and alter the membrane permeability. So, due to the presence of three types of flavonoids in the extract of this plant, it shows antibacterial activity [60].

Other studies have reported different species of the *Scrophularia* as a rich source of alkaloids, resin glycosides, iridoid glycosides, mainly aucubin and catalpol which are usually are found in different parts of plants like as leave, skin, stem, bud and scion. Iridoids are a large group of cyclopentan-[c]-pyran monoterpenoids which are responsible for biological activity of *Scrophularia* spp. extract including antibacterial activities. These natural compounds that are widely employed for medicinal purposes can inhibit bacterial growth and division as well as inhibit biofilm formation [61, 62]. Alkaloids have antibacterial activity as they can disrupt the bacterial cell membrane, affect the DNA function and inhibit protein synthesis [63].

#### Investigating the antibacterial activity of nanofibers prepared by disc diffusion method

Figure 9; Table 4 show the results of the maximum diameter of the halo growth inhibition zone of zein nanofibers containing extract against Gram-positive and Gram-negative bacteria. It can be concluded that the antibacterial properties of the nanofibers in disc diffusion method were most effective against the Gram-negative bacteria *Escherichia coli and Pseudomonas aeruginosa*. In comparison with antibacterial results of the extract, it can be concluded that zein nanofiber has increased antibacterial potential of this extract against Gram-negative bacteria. It can be due to its higher diffusion rate across the outer membrane and hence promoted the antibacterial potential of extract. However, these nanofibers were ineffective against *Bacillus subtilis*. Possibly size increasing has limited the accessibility of zein nanofiber to its final target and hence no growth inhibition was found.

**Table 4:** Results of the antibacterial activity of zein nanofibers containing extract

Bacterial species	Diameter of growth inhibition (mm)
*Bacillus subtilis*	-
*Staphylococcus aureus*	17
*Pseudomonas aeruginosa*	28
*Escherichia coli*	27

#### Investigating the antibacterial activity of prepared nanofibers in broth culture

The absorbance of the prepared samples was read and the obtained results are shown in Fig. 10. The results showed that zein nanofibers containing maceration extract were capable of inhibiting the growth of four tested bacteria. This effect can be due to the release of the extract in the liquid medium, and thus its antibacterial effect was well observed in the broth culture [64].

#### Colony count

Colony count is a method for enumeration of live and active bacteria. During treatment of bacterial cultures with antibacterial compounds, the total formed colony will be reduced [65]. Two types of *Escherichia coli* bacteria (gram negative) and *Bacillus subtilis* (gram positive) were tested. Results of colony count of bacteria treated with zein nanofibers are shown in Table 5. By comparing the number of colonies of the control group and nanofibers, it can be seen that the fibers had the ability to reduce the number of living bacteria. As can be found from the Table 5, zein nanofibers containing extract showed more inhibitory effect against *E. coli* as Gram-negative species compared to B. subtilis as Gram-positive species while the extract had showed less inhibitory effect against Gram-negative species. The less sensitivity of Gram-negative bacteria to this extract may be due to their outer membrane structure that limits accessibility of active compounds to their target in bacterial cells. However, it seems its composition with zein enhances its diffusion through the outer membrane and hence more inhibitory effect was created against Gram-negative bacteria. The percentage of colony reduction was calculated using Eq. 1 and reported in Table 6.

1 $$\:\text{C}\text{o}\text{l}\text{o}\text{n}\text{y}\,\text{p}\text{e}\text{r}\text{c}\text{e}\text{n}\text{t}\,\text{r}\text{e}\text{d}\text{u}\text{c}\text{t}\text{i}\text{o}\text{n}=\frac{Colony\:number\:of\:control\:samples\_\:Colony\:number\:of\:the\:desired\:sample}{Colony\:number\:of\:control\:samples}\times\:100$$

**Table 5:** Colony count bacterial suspension treated with zein nanofibers containing maceration extract

**Type of composition**	**Bacteria species**	**Colony number of primary suspension (CFU mL**^−1^**)**
Zein nanofibers containing extract	*Bacillus subtilis*	1.6 × 10^8^
	*Escherichia coli*	1.8 × 10^8^
Control group	*Bacillus subtilis*	4.25 × 10^8^
	*Escherichia coli*	3 × 10^9^

**Table 6:** Colony reduction percentage of the primary suspension of zein nanofibers containing ethanolic maceration extract

Composition	Colony reduction (%)	
	* Bacillus subtilis *	* Escherichia coli *
Zein nanofibers containing extract	62.35	94

#### Release studies

The presence of biophenols in the plant extract, due to their antioxidative activity, has made the nanofibers containing the extract as an active mat against bacterial growth [66]. The biophenol contents of the ethanolic maceration extract of the *Scrophularia striata* plant were measured by the Folin-Ciocalteu reagent. The results showed that this extract has the highest biophenolic content of 79.8 ± 0.6 mg g^−1^ (*n* = 3) compared to the extracts prepared by Soxhlet and ultrasonic methods. These outcomes agree with the antibacterial tests, which showed that the ethanolic maceration extract has a great ability to inhibit the growth of bacteria.

Then, the release rate of biophenol components from zein nanofibers/*Scrophularia striata* scaffold was investigated by the Folin-Ciocalteu reagent in the PBS solution (pH = 7.4) and water. The PBS solution containing nanofibers without extract showed a slight blue color upon the addition of Folin’s reagent, indicating the presence of small amounts of biophenol in these nanofibers [67]. Therefore, in order to determine the amount of biophenol released from the extract loaded in the nanofibers, the content of biophenol measured in the zein fibers without extract was subtracted from the amount of biophenol obtained in the nanofibers containing the extract [68]. Finally, the amount of biophenol released from the extract in PBS medium was obtained and its variation was studied over time (Fig. 11). In order to investigate the role of the environment in the release of effective substances, all the above experiments were also carried out for nanofibers in the water. The results show that the biophenol released from nanofibers containing maceration extract of *Scrophularia striata* in a PBS solution was about 2 times greater than the released biophenol in the water, which is considered an advantage for nanofibers containing extract. Because the PBS solution (pH = 7.4) has the same type and concentration as the biological environment of the human body, and nanofibers can release more effective substances to promote wound healing when used as a wound coating [30]. Additionally, the release of active substances initially shows an increase up to 3 h, followed by gradual changes, although this trend is more regular in the PBS environment [69]. In Fig. 11 comparison of the release of biophenol components in two environments, PBS and water, is presented.

### Biocompatibility and biodegradability

#### Examining the toxicity of the extracts on cells by MTT method

To check the level of cytotoxicity, MTT colorimetric test was performed on fibroblast cells. In.

the MTT method, cell viability is determined based on the activity of mitochondrial succinate dehydrogenase enzymes [70]. Using Eq. 2, the survival rate of each sample was calculated. This test was performed for the maceration extracts of the plant [71]. Figure 12 shows that the cell survival rate for the maceration extract of the *Scrophularia Striata* plant at a concentration of 0.01 mg mL^−1^ is 100%, while at a concentration of 10 and 50 mg mL^−1^, cell survival is significantly reduced. Overall, with the increase in the concentration of the extract, cell viability has decreased, and the percentage of toxicity has increased.

2 $$\:\text{C}\text{e}\text{l}\text{l}\:\text{s}\text{u}\text{r}\text{v}\text{i}\text{v}\text{a}\text{l}\:\text{r}\text{a}\text{t}\text{e}=\frac{Optical\:absorbance\:of\:treated\:cells}{Optical\:absorbance\:of\:control\:cells}\:\times\:100$$

#### Evaluation of cell survival on zein nanofibers without extract and containing extract by the MTT method

Figure 13 shows that in the presence of zein nanofibrous scaffolds containing extract, the survival of fibroblast cells is 91% after 24 h and 92% after 48 h, while the cell survival in the presence of zein nanofibrous scaffolds without extract is 84 and 86%, respectively, after the same time periods [72]. Therefore, cell survival in the presence of nanofibers containing the extract is significantly higher compared to the presence of the control nanofiber scaffold [73].

The reason for this result can be attributed to the tendency for greater degradation of scaffolds without extract, as these scaffolds degrade more quickly in culture media and their release leads to cellular degradation sooner than that of scaffolds containing extract, which have a slower degradation rate. It can also be concluded that an appropriate concentration of extract has been added to the zein nanofibers. Also, this increase in cell survival can be attributed to the positive role of the extract of the plant, which promotes the proliferation of fibroblast cells in a concentration-dependent manner and at low concentrations (concentrations less than 0.01 mg mL^−1^). Although, in general, both types of scaffolds were suitable for the cultivation of fibroblast cells [74].

#### In vivo evaluation of nanofibers wound dressing

**Fig. 15:**
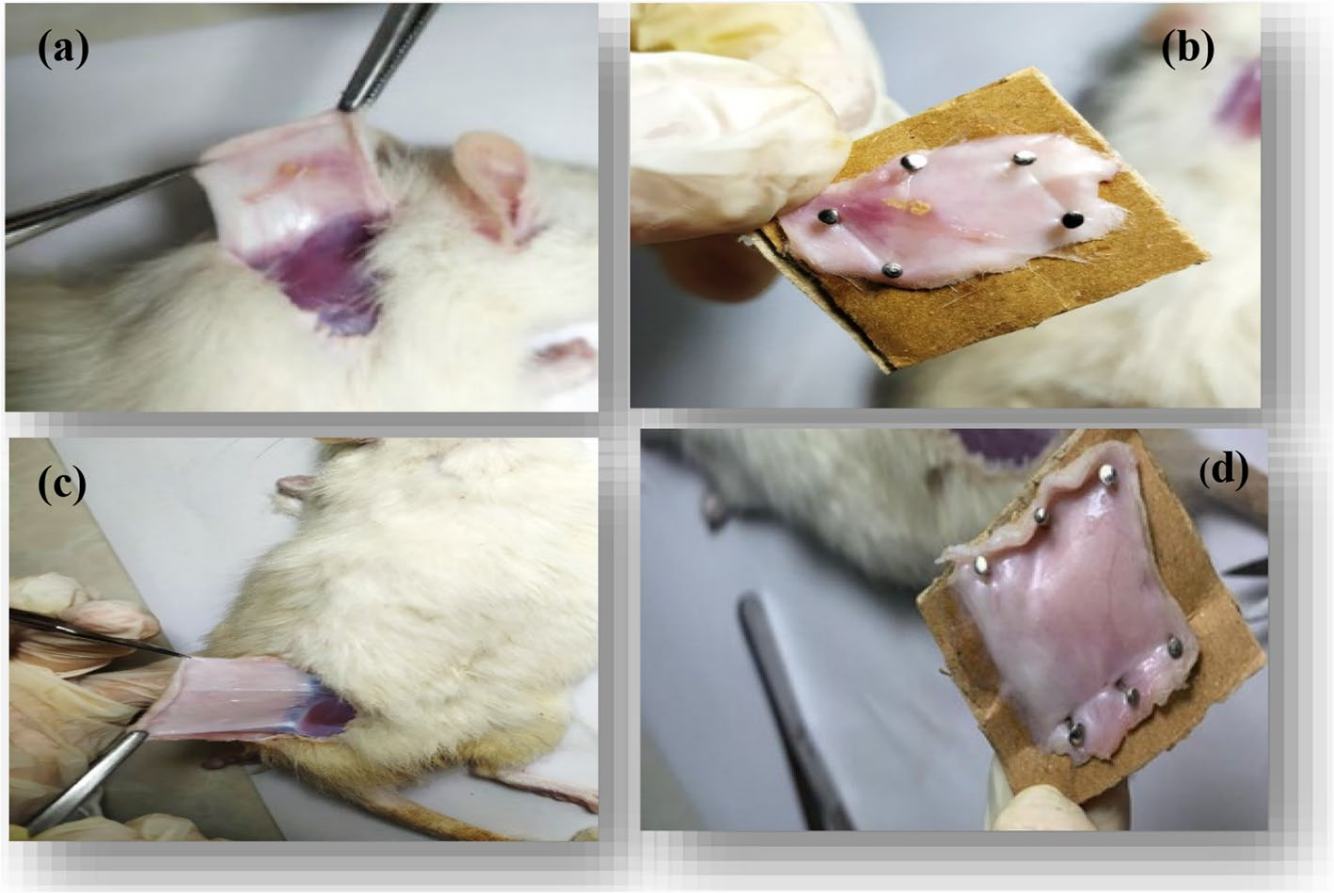
Image (**a**) partial presence of nanofibers without extract after one month under the skin in the neck area of mice. **b** Preparation of skin tissue isolated from the neck area along with nanofibers without extract for histomorphology tests. **c** Opening the skin of a part of the mouse’s back that was implanted with optimal nanofibers after one month. **d** Preparation of skin tissue isolated from the lumbar region for histomorphology experiments

In Fig. 14, macroscopic examinations revealed that both the control nanofibers and the nanofibers containing the extract remained distinct and identifiable after two weeks. No inflammation was observed around them, and the mice were in excellent health. It is noteworthy that a significant amount of tissue was formed on the nanofibers containing extract, whereas the amount of tissue formed on the control nanofibers was less. After four weeks, the mice in the other group were euthanized, and the skin on their necks and backs was removed and evaluated as in the previous stage. As shown in Fig. 15, in the neck area (parts (a) and (b)) where the control nanofibers were located, residues of the scaffold have remained on the skin. In the back region (Parts (c) and (d)), few residues of the nanofibers containing extract were observed. Additionally, no inflammation was observed in either the back or neck areas.

#### In vivo and transplantation studies

**Fig. 16:**
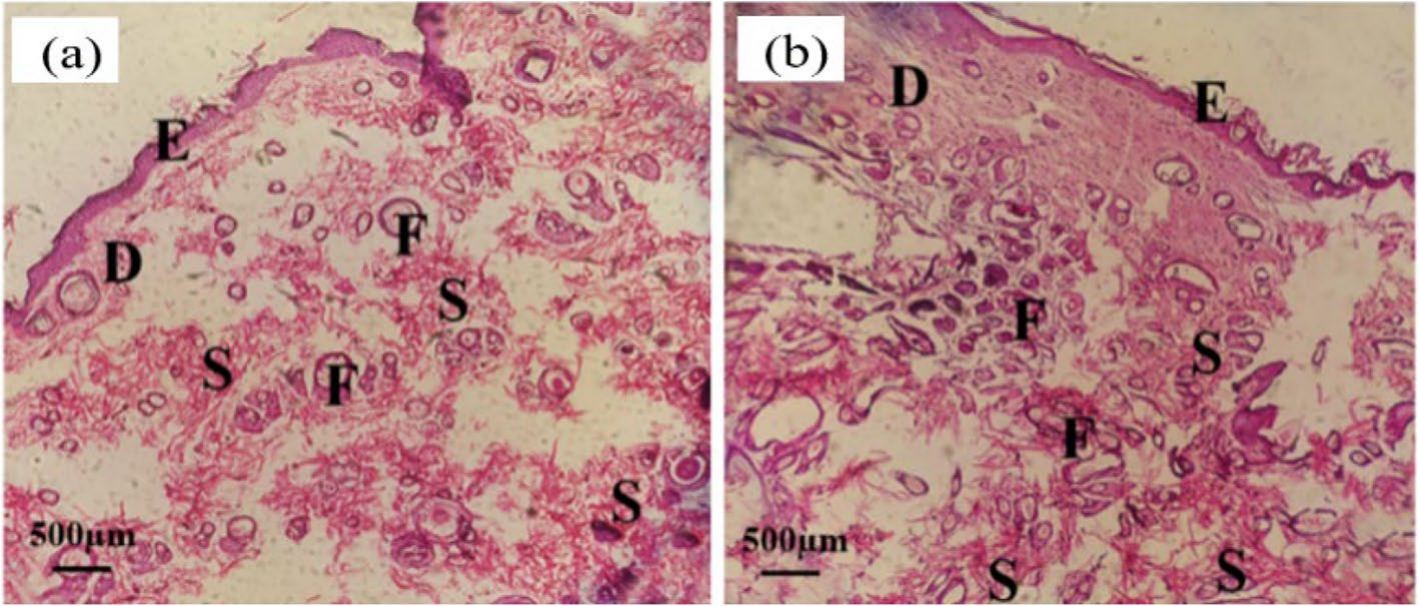
H & E staining of (**a**) zein nanofibers without extract (control) after two weeks of implantation, (**b**) zein nanofibers containing extract (optimum) after two weeks of implantation

**Fig. 17:**
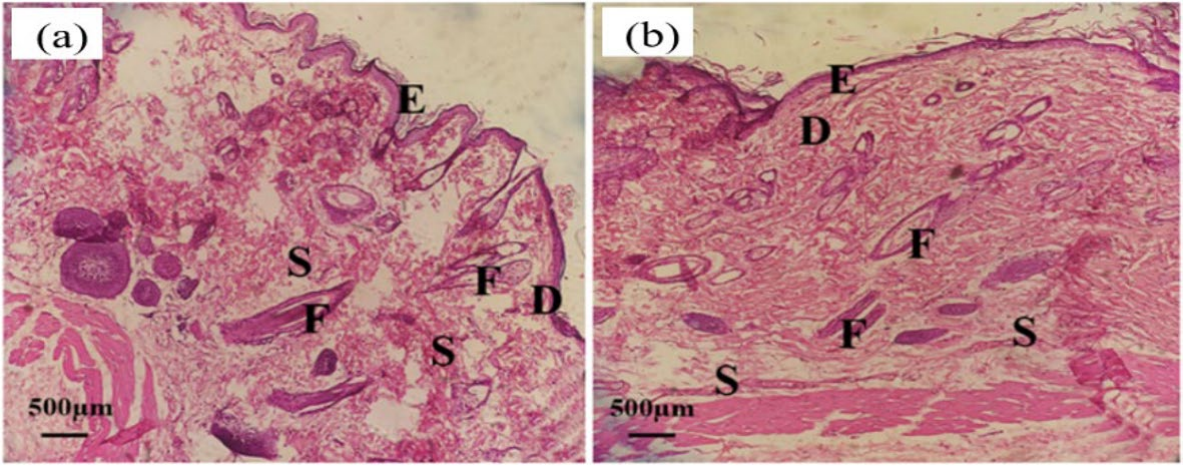
H & E staining related to (**a**) zein nanofibers without extract (control) after one month of planting, (**b**) zein nanofibers containing extract (optimal) after one month of planting

The histomorphological images obtained from hematoxylin-eosin (H&E) staining of skin sections for nanofiber grafting are shown after two weeks in Fig. 16 and after one month in Fig. 17. In both pictures, E represents the epidermal ridges, D represents the dermis, F represents the hair follicles, and S is the remnants of nanofibers.

In Fig. 16, H&E staining reveals remnants of nanofibers after two weeks, with empty spaces indicating nanofiber degradation. Considering the H&E staining in Fig. 17(b) after one month, obtained from the implantation of zein nanofibers containing extract, only remnants of the scaffold are visible. More dermis has formed, there is an increased presence of connective tissues, the number of follicles has also increased, better healing has occurred, more blood vessels, and the tissue has become denser. Conversely, in part (a) representing zein nanofibers without extract, voids are still present, indicating ongoing nanofiber degradation. Overall, H&E staining after two weeks and a month post-implantation revealed no inflammatory cells in the tissue sections, indicating the absence of inflammation and infection. Thus, both zein nanofibers without extract and those containing extract exhibited acceptable biocompatibility in in vivo condition.

#### Future prospects

Future research on nanofibers containing *Scrophularia striata* extract should focus on several key areas. Investigating mixtures of *Scrophularia striata* extract with other natural extracts which have complementary properties and investigating their synergistic effect should be considered. Research on the long-term efficacy and safety of the nanofibers on different types of wounds and in different populations is also important to confirm their biocompatibility and effectiveness. Furthermore, the extract of *Scrophularia striata* can also be encapsulated in hydrogels, and its effect can be compared with that of nanofibers.

The production and adoption of electrospun nanofiber dressings face several challenges, including scalability in manufacturing, regulatory hurdles, and the need to educate both physicians and patients about the advantages of these dressings over traditional methods. These nanofibers have several applications, including medical applications, particularly in wound healing, drug delivery, and tissue engineering. Also, their antimicrobial properties help prevent infections.

## Conclusions

This study presents a biocompatible scaffold prepared of zein nanofibers incorporated with ethanolic maceration extract of *Scrophularia striata* as wound dressing by a one-step electrospinning technique. Under optimum electrospun conditions, zein nanofibers with bead-free and uniform morphology were successfully produced. The addition of extract reduced the diameter of nanofibers while maintaining their uniform structure. FT-IR spectra confirmed the presence of the extract within the nanofibers tissue, and elemental mapping analysis demonstrated a uniform distribution of elements within the nanofibers. Water contact angle determination also proved that nanofibers containing extract are more hydrophilic compared to those without extract which maintains an adequate moist wound environment, conducive to wound healing.

Hospital and/or community acquired infections are the main consequences of wound healing which in some cases may lead to death as a result of infection with antibiotic resistant bacteria. Improper wound dressing and its long-term remaining are causes of such infections. The prepared electrospun zein nanofibers containing *Scrophularia striata* extract suggest a slow-release dressing with potent antibacterial properties that can reduce wound infections. Interestingly, this mat showed a bactericidal effect against *P. aeruginosa* which is a common cause of wound infections.

Furthermore, the MTT test revealed higher cell survival rates in the presence of nanofibers containing the extract than those without it. Biocompatibility was assessed in vivo by implanting nanofibers into the bodies of mice. Subsequent macroscopic and microscopic examinations revealed no signs of inflammation. H&E staining images also demonstrated improved tissue repair at the site of nanofibers implantation, with no signs of infection or inflammatory cells, indicating the biocompatibility of the nanofibers.

## Data Availability

The data that support the findings of this study are available from the corresponding author upon reasonable request.
